# Inspiratory and expiratory CT analyses of the diaphragmatic crus in chronic obstructive pulmonary disease

**DOI:** 10.1007/s11604-022-01314-w

**Published:** 2022-07-12

**Authors:** Shinji Wada, Shin Matsuoka, Hidefumi Mimura

**Affiliations:** Department of Radiology, St. Marianna University School of Medicine, 2-16-1, Sugao, Miyamae-ku, Kawasaki City, Kanagawa Japan

**Keywords:** Chronic obstructive pulmonary disease, Diaphragmatic crus, Inspiratory and expiratory CT

## Abstract

**Purpose:**

This study aimed to investigate the association between the results of pulmonary function tests (PFTs) in patients with chronic obstructive pulmonary disease (COPD) and the size of their diaphragmatic crus (DC) using inspiratory and expiratory CT.

**Materials and methods:**

Thirty-three patients who underwent inspiratory and expiratory CT and PFTs between July and December 2019 were studied retrospectively. The short axis, long axis, and cross-sectional area (CSA) of the bilateral DC were measured, and the percentage change of the DC after expiration (% change of DC) in the size was calculated. The correlation between the results of the PFTs (forced expiratory volume in 1 s [FEV_1_], FEV_1_/forced vital capacity [FVC], and percent predicted FEV_1_ [%FEV_1_]) and the size and % change of DC was statistically analyzed.

**Results:**

Significant correlations were observed between the short axis of the right and left DC at expiration and PFTs (FEV_1_, *r* = –0.35, –0.48, *p* = 0.04, .007; FEV_1_/FVC, *r* = –0.52, –0.65, *p* = 0.002, < .001; %FEV_1_, *r* = –0.56, –0.60, *p* < 0.001, < 0.001; respectively), between the CSA of the right DC at expiration and PFTs (FEV_1_/FVC, *r* = –0.42, *p* = 0.01; %FEV_1_, *r* = –0.41, *p* = 0.017; respectively), and between the % change of the short axis of the left DC and the CSA of the left DC and PFTs (FEV_1_, *r* = 0.64, 0.56, *p* < 0.001, .001; %FEV_1_, *r* = 0.52, 0.51, *p* = 0.004, 0.004; respectively). The smaller the short axis of the DC and CSA at expiration and the larger the % change in DC of the CSA, the lower the airflow limitation.

**Conclusion:**

There were significant correlations between airflow limitation and the short axis of the bilateral DC at expiration, and the % change in the DC of the CSA. Certain CT measurements of the DC may reflect airflow limitation in patients with COPD.

**Supplementary Information:**

The online version contains supplementary material available at 10.1007/s11604-022-01314-w.

## Introduction

Chronic obstructive pulmonary disease (COPD) is a common, preventable, and treatable disease that is characterized by persistent respiratory symptoms and airflow limitation and is currently one of the top three causes of death worldwide [1, 2]. Skeletal muscle disorder, especially limb muscle dysfunction, is an important systemic consequence of COPD because of its impact on physical activity, exercise tolerance, quality of life, and even survival in this disease [3]. In patients with COPD, the diaphragm becomes weak and unable to function as it should when breathing; it is thought that this is due to changes in the muscle cells of the diaphragm, causing the muscle fibers to lose the strength needed to contract and relax [4]. Previous reports have shown that CT-based measurements of the cross-sectional area (CSA) of the intercostal and abdominal [5], mid-thigh [6], pectoralis [7], and erector spinae muscles [8] were associated with exacerbations and prognosis of patients with COPD.

The diaphragm is the main respiratory muscle, and although it regulates respiratory function [9], there are few studies on diaphragm morphology. It is hypothesized that this is because the diaphragm has a complicated 3D dome shape, and it changes with inspiration and expiration. Therefore, it is difficult to measure the entire shape of the diaphragm. Measurements of the diaphragm thickness with ultrasonography [10, 11] and 3D CT quantification of the diaphragm [12–14] have also been attempted; however, these measurements are time-consuming. We focused on the diaphragmatic crus (DC), which is a part of the diaphragm that can be easily measured on cross-sectional CT images. In fact, the DC is where the diaphragm attaches to the vertebral body, and several studies have evaluated the DC using CT [15–18]. Donovan et al. demonstrated that CT-assessed diaphragm morphology was associated with COPD severity [14]; however, they evaluated diaphragm morphology only on inspiratory CT images. Since the diaphragm changes with inspiration and expiration, it is not clear which respiratory phase of diaphragm morphology is associated with airflow limitation in patients with COPD. We hypothesized that the size of the DC during either inspiration or expiration, or the rate of change in DC size and CSA with respiration, would be associated with airflow limitation. This study aimed to evaluate the association between the size of CT measurements of the DC in both inspiratory and expiratory CT and airflow limitation and to determine which of the inspiratory or expiratory diaphragm morphology is more associated with airflow limitation in patients with COPD.

## Materials and methods

### Materials

This retrospective study was approved by our institutional review board (5307), which waived the need for informed consent. Inspiratory and expiratory CT scans were performed between July and December 2019 in 37 consecutive patients with COPD who underwent pulmonary function tests (PFTs) within 1 week of obtaining CT scans. The clinical indications for CT varied and included the detection or routine observation of emphysema. Patients with COPD classified as Global Initiative for Chronic Obstructive Lung Disease Stages I to IV were included and divided into emphysema-dominant and chronic bronchiolitis-associated types [1]. The exclusion criteria were presence of lung tumors and previous lung, chest wall, or upper abdominal surgery. Four patients were excluded for the following reasons: lung cancer (*n* = 1), post-mastectomy (*n* = 1), post-total gastrectomy (*n* = 1), and post-pneumonectomy (*n* = 1). Thus, 33 patients (median age, 75 [range, 41–88] years; 29 men and 4 women) were included in this study. Patient characteristics are summarized in Table 1. We calculated that the minimum sample size needed to detect a correlation (*r*) of 0.5 between the parameters of the DCs and PFTs with 80% power and a significance level of 0.05 would be 30 patients. Therefore, our sample size of 33 patients was considered sufficient for the detection of such correlations.

**Table 1:** Patient characteristics

Variable	Data
Total number of patients	33
Age, year	72.7 ± 9.37 (41–88)
Sex, M/F	29/4
Height, m	1.63 ± 0.07 (1.47–1.77)
Weight, kg	58.3 ± 8.10 (45–80)
BMI, kg/m^2^	21.94 ± 2.90 (18.28–31.16)
Smoking history, pack-years	51.67 ± 32.69 (0–126)
GOLD stage, I/II/III/IV	7/14/9/3
Subtype of COPD, emphysema/chronic bronchiolitis	31/2
VC, L	3.24 ± 0.68 (1.62–4.58)
FEV1, L	1.54 ± 0.57 (0.5–2.57)
FEV1/FVC, %	49.01 ± 13.33 (23.57–68.46)
FEV1, % predicted	60.67 ± 22.43 (15.55–105.06)

Data are reported as mean ± standard deviation

*M* male, *F* female, *BMI* body mass index, *GOLD* global initiative for chronic obstructive lung disease, *COPD* chronic obstructive pulmonary disease, *VC* vital capacity, *L* left, *FEV1* forced expiratory volume in 1 s, *FVC* forced vital capacity

### CT scan protocol

All patients underwent CT scans using a 64-detector CT scanner (Aquilion-64; Canon Medical, Tokyo, Japan). The scanner was regularly calibrated with air and a water phantom to allow for reliable measurements. CT was performed during a breath-hold in both deep inspiration and expiration with the patient in the supine position. Before scanning, each patient was carefully instructed on how to breathe during scanning. Multislice CT parameters for both CT studies were as follows: collimation, 0.5 mm; 120 kV; 100 mA at inspiration; 40 mA at expiration; gantry rotation time, 0.5 s; and beam pitch, 0.83 (table feed per gantry, 53 mm; collimation beam width, 64 mm). All images were reconstructed using a standard reconstruction algorithm with a section thickness of 1 mm and a reconstruction interval of 0.5 mm. The voxel size was 0.625 × 0.625 × 1 mm. In this study, we adopted reconstructed images with 5-mm thickness to minimize errors by each measurer.

### CT measurement of the DC

**Fig. 1:**
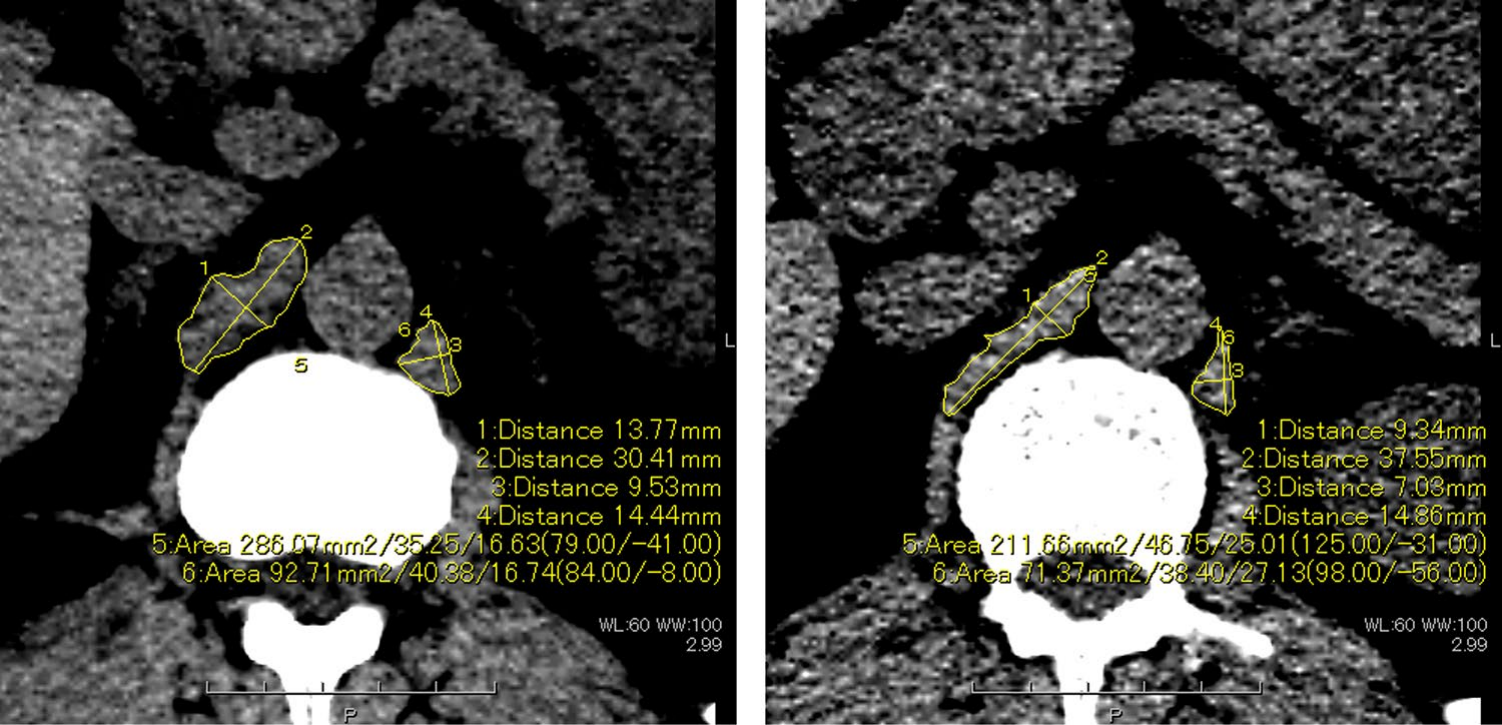
Measurements of the diaphragmatic crura at inspiratory and expiratory CT using a commercially available interpretation viewer. The left figure presents the cross-sectional image at inspiration, and the right figure presents the cross-sectional image at expiration at the orifice of the superior mesenteric artery under the mediastinal window

In the cross-sectional CT image of 5-mm thickness under the mediastinal window, the left and right DC were measured at the height of the origin of the superior mesenteric artery, referring to a previous report [16], using a commercially available interpretation viewer (Canon Medical). The window level and width were set to 60 HU and 100 HU, respectively, to clarify the boundaries between the DC and surrounding fat and to facilitate measurement. The short and long axes of both sides of the DC were measured manually. The CSA of the bilateral DC was calculated by manually surrounding the DC with a region of interest (Fig. 1).

### Reproducibility of measuring the DC

Regarding reproducibility, the DC was measured at both inspiratory and expiratory CT determined in 20 randomly selected subjects by two authors and assessed using Bland–Altman analysis [19]. All DC sizes were calculated by two certified diagnostic radiologists.

### PFTs

PFTs were performed within 1 week of obtaining CT scans. The forced expiratory volume in 1 s (FEV_1_) and forced vital capacity (FVC) were measured using standard techniques [18], and the ratio of FEV_1_ to FVC (FEV_1_/FVC) was determined. Values for each PFT, except for FEV_1_/FVC, were expressed as percentages of predicted values according to prediction equations described elsewhere [20].

### Statistical analysis

Normalized variables are shown as mean ± standard deviation, and non-normalized variables are shown as median (interquartile range). The association between the size of the DC and the results of PFTs were assessed using simple linear regression analysis followed by multiple regression analysis to adjust for age, sex, body mass index, and smoking per pack-years. Statistical analysis was performed using JMP software (version 14.2.0; SAS Institute, Cary, NC). Statistical significance was established at *p* < 0.05.

## Results

### Validation of measurements

Plots of the mean of and the difference between measurements of the left short axis of the DC at inspiration, which were used to assess reproducibility, are shown in Supplemental Fig. 1. In the reproducibility study, the mean difference was 0.047 mm, and the limit of agreement was –0.1831 to 0.08905. In each of the other plots, the mean difference did not appreciably deviate from zero, and the limits of agreement were small.

### Measurements of sizes and change rates of DCs at inspiratory and expiratory CT

Measurements of DC size on inspiratory and expiratory CT scans and correlations with the results of PFTs are shown in Table 2. In three male patients (two with emphysema type and one with chronic bronchiolitis type), the left DC was outside of the measured cross-section. Therefore, their left DC was assumed to be the missing value.

**Table 2:** Measurements of the diaphragmatic crura at inspiratory and expiratory CT and correlations with results of pulmonary function tests

Diaphragmatic crus				FEV1		FEV1/FVC		FEV1, % predicted	
				*r* value	*p* value	*r* value	*p* value	*r* value	*p* value
At inspiration	Short axis	R	10.31 ± 2.41 mm	– 0.12	0.51	– 0.3	0.09	– 0.28	0.12
		L	6.89 [6,25, 9.04] mm	0.05	0.78	– 0.23	0.23	– 0.15	0.42
	Long axis	R	30.35 ± 6.96 mm	0.14	0.42	0.05	0.8	0.04	0.8
		L	15.82 ± 6.78 mm	0.41	0.0256^†^	0.24	0.2	0.29	0.12
	CSA	R	210.02 ± 67.6 mm^2^	– 0.02	0.91	– 0.16	0.38	0.16	0.37
		L	83.97 ± 47.14 mm^2^	0.34	0.06	0.1	0.59	0.19	0.3
At expiration	Short axis	R	7.95 ± 1.89 mm	– 0.35	0.0444^†^	– 0.52	0.0018^†‡^	– 0.56	0.0007^†‡^
		L	6.04 ± 2.15 mm	– 0.48	0.0066^†‡^	– 0.65	0.0001^†‡^	– 0.6	0.0004^†‡^
	Long axis	R	32.03 ± 6.66 mm	0.06	0.73	– 0.06	0.72	– 0.06	0.73
		L	14.12 [11.15, 20.70] mm	0.37	0.0453^†^	0.22	0.25	0.27	0.14
	CSA	R	170.50 ± 55.20 mm^2^	– 0.26	0.14	– 0.42	0.0141^†‡^	– 0.41	0.0173^†‡^
		L	70.05 ± 35.11 mm^2^	0.08	0.67	– 0.15	0.41	– 0.06	0.74

Normally distributed data are reported as mean ± standard deviation, and non-normalized data are reported as median [interquartile range] and range in parentheses

*R* right, *L* left, *CSA* cross-sectional area, *FEV1* forced expiratory volume in 1 s, *FVC* forced vital capacity

^†^*P* < 0.05, data unadjusted analysis

^‡^*P* < 0.05, data adjusted analysis

The percentage change in the size of the DC after expiration (% change of DC) and correlations with the results of PFTs are shown in Table 3. In the right DC, the short axis was extended by 32%, the long axis was contracted by 6%, and the CSA was 21% larger during inspiration than during expiration. In the left DC, the short axis was barely extended, the long axis was contracted by 5%, and the CSA was 20% larger during inspiration than during expiration.

**Table 3:** Measurements of rate of change in the size of the diaphragmatic crus due to respiration and correlations with results of pulmonary function tests

Diaphragmatic crus			FEV1		FEV1/FVC		FEV1, % predicted	
		Rate of change due to respiration	*r* value	*p* value	*r* value	*p* value	*r* value	*p* value
Short axis	R	1.32 ± 0.25	0.24	0.19	0.25	0.16	0.32	0.07
	L	1 [0.92, 1.52]	0.64	0.0002^†‡^	0.48	0.0078^†‡^	0.52	0.0035^†‡^
Long axis	R	0.94 [0.86,1.00]	0.14	0.43	0.23	0.2	0.2	0.26
	L	0.95 [0.83, 1.06]	– 0.02	0.94	– 0.01	0.94	– 0.04	0.84
CSA	R	1.21 [1.06, 1.38]	0.39	0.0232^†‡^	0.46	0.0065^†‡^	0.44	0.0096^†‡^
	L	1.20 ± 0.29	0.56	0.0014^†‡^	0.44	0.014^†‡^	0.51	0.0043^†‡^

Normally distributed data are reported as mean ± standard deviation, and non-normalized data are reported as median [interquartile range] and range in parentheses

*R* right, *L* left, *CSA* cross-sectional area, *FEV1* forced expiratory volume in 1 s, *FVC* forced vital capacity

^†^*P* < 0.05, data unadjusted analysis

^‡^*P* < 0.05, data adjusted analysis

### Correlation between the size of DC and PFTs

**Fig. 2:**
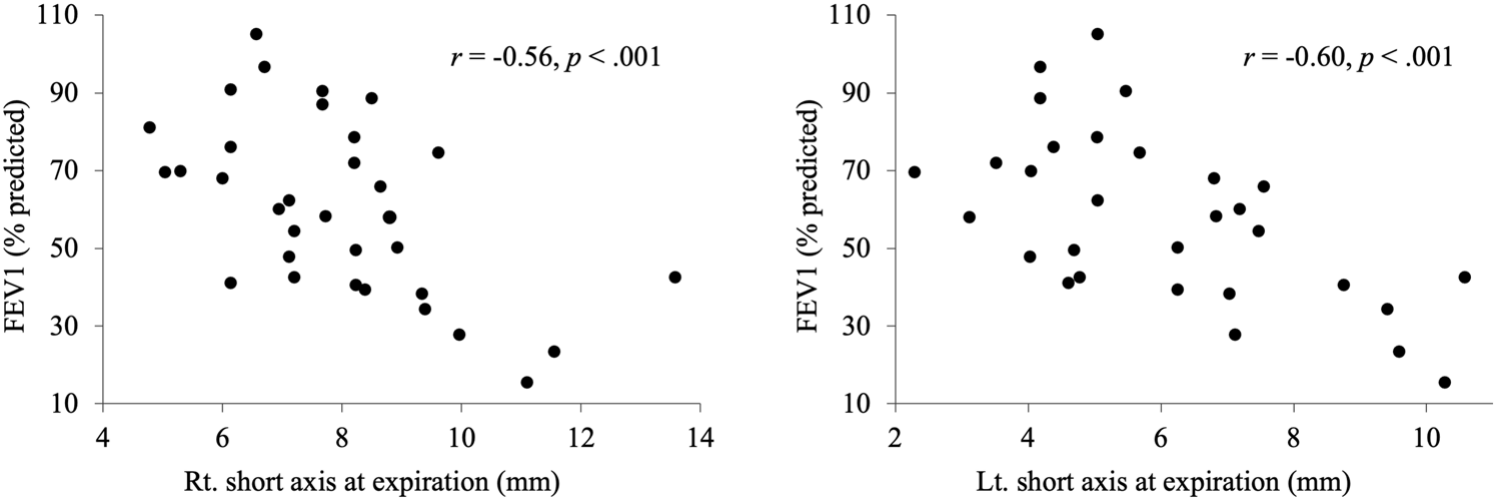
Negative correlation between the short axis of the bilateral diaphragmatic crura at expiration and pulmonary function tests

There were significant negative correlations between the short axes of the right and left DC at expiration and PFTs (FEV_1_, *r* = –0.35, –0.48, *p* = 0.04, 0.007; FEV_1_/FVC, *r* = –0.52, –0.65, *p* = 0.002, < 0.001; %FEV_1_, *r* = –0.56, –0.60, *p* < 0.001, < 0.001; respectively) and between the CSA of the right DC at expiration and PFTs (FEV_1_/FVC, *r* = –0.42, *p* = 0.01; %FEV_1_, *r* = –0.41, *p* = 0.017; respectively) (Fig. 2). The adjusted analysis also showed significant correlations between the left and right short axes or right CSA of the DC at expiration and FEV_1_/FVC or %FEV_1_ (Table 2). A larger short axis of the DC and larger CSA at expiration were associated with stronger airflow limitation.

### Correlation between the % change of DC due to respiration and PFTs

There was a positive correlation between the % change in DC of the short axis of the left DC and the CSA of the right and left DC and PFTs (FEV_1_, *r* = 0.64, 0.56, *p* < 0.001, 0.001; %FEV_1_, *r* = 0.52, 0.51, *p* = 0.004, 0.004; respectively). The adjusted analysis showed similar significant differences (Table 3). The larger the % change of bilateral DC in the CSA (i.e., the CSA of the DC at inspiration was greater than at expiration), the less airflow limitation was observed (Supplemental Fig. 2).

## Discussion

In this study, we found that a smaller short axis of the DC and CSA at expiration and a larger % change in DC of the CSA correlated with airflow limitation in patients with COPD. To the best of our knowledge, this is the first report to show the correlations between the size of the DC measured using both inspiratory and expiratory CT and the results of PFTs in patients with COPD.

There have been several studies on the correlation between the size of the diaphragm and the results of PFTs and various other medical conditions [10–14]. Donovan et al. [14] showed that CT-assessed diaphragm morphology was associated with COPD severity, exacerbations, impaired health status, and exercise intolerance. In our study, there was also a correlation between the size of the DC at expiration or the % change of DC in the CSA and the results of PFT in patients with COPD. We found that the DCs were thicker and the CSA was larger at inspiration. In fact, the greater the % change of DC in the CSA, better PFT results were maintained. The results of this study show that it is useful to evaluate both expiratory and inspiratory CT images from the perspective of estimating pulmonary function. Donovan et al. [14] demonstrated that evaluating only inspiratory CT is also relevant, and they also considered certain parameters, such as the CT value of the diaphragm. However, it is difficult to compare the results of the entire diaphragm with those of the DC.

There are several studies on the image quantification of the DC itself; the right DC is generally longer and thicker than the left DC, and the DC is thicker on inspiration than on expiration [16]. This study showed similar results in patients with COPD, and there were significant correlations between larger changes in the CSA at the origin of the superior mesenteric artery during respiration and the results of PFTs. As the position of the attachment part of the DC is fixed, the diaphragm is lowered by inspiration; when the DC is evaluated in a cross-sectional image, it becomes thicker and enlarges to a short axis, and the CSA is larger at inspiration than that at expiration. The fact that the size of the DC during expiration correlates more strongly with airflow limitation than during inspiration may reflect the difficulty of extending the DC due to an expiratory limitation caused by COPD.

The diaphragm is an important respiratory muscle, but it is not clear how involved the DC is in respiratory physiology. It is also unclear whether the DC is the part of the diaphragm that best reflects the organization of COPD, as there is a report of different collagen content and distribution of abnormal muscle fibers in different parts of the diaphragm in COPD donors [21]. However, it may be clinically useful to be able to estimate some aspects of respiratory function by measuring the DC on inspiratory and expiratory chest CT.

This study has some limitations. First, the number of patients was small in this retrospective study. The position of the origin of the DC is slightly different between the left and right sides, and the DC at the origin of the superior mesenteric artery was measured with a 5-mm thickness, referring to a previous study [16]. However, the optimal position for measuring the DC is unclear. In addition, changes in the DC cranio-caudal configuration cannot be ignored, and inspiratory and expiratory scans may not always be able to measure the same area. Furthermore, inspiratory and expiratory CT scans were not performed on non-COPD patients; therefore, it was not possible to evaluate the association between the size and % change of DC and airflow limitation in these patients.

The amount of radiation exposure is a limitation of the technique used for paired inspiratory and expiratory CT. However, the latest-generation CT system has a lower risk of cancer without compromising image quality at a lower radiation dose, even when taking inspiratory and expiratory images, as compared with the inspiratory-only scan with an older CT system [22]. We also consider that a further radiation dose reduction is achievable using deep-learning reconstruction [23] and iterative reconstruction techniques [24].

In conclusion, there were significant correlations between the short axis of the bilateral DC at expiration, % change in DC of the CSA, and airflow limitation parameters derived from PFT. These results show that inspiratory and expiratory CT measurements of the DC can be used as markers for COPD.

## Supplementary Information

Below is the link to the electronic supplementary material.

Supplementary file1 (DOCX 23013 KB)
