# Supplementary material for: Inspiratory and expiratory CT analyses of the diaphragmatic crus in chronic obstructive pulmonary disease
Source: Jpn J Radiol. 2022 Jul 12;40(12):1257–62. doi: 10.1007/s11604-022-01314-w (PMC9719885; doi:10.1007/s11604-022-01314-w)
Supplement: Supplementary file 1 — Supplementary file1 (DOCX 23013 KB) [file 11604_2022_1314_MOESM1_ESM.docx]

**Supplemental Material**

**
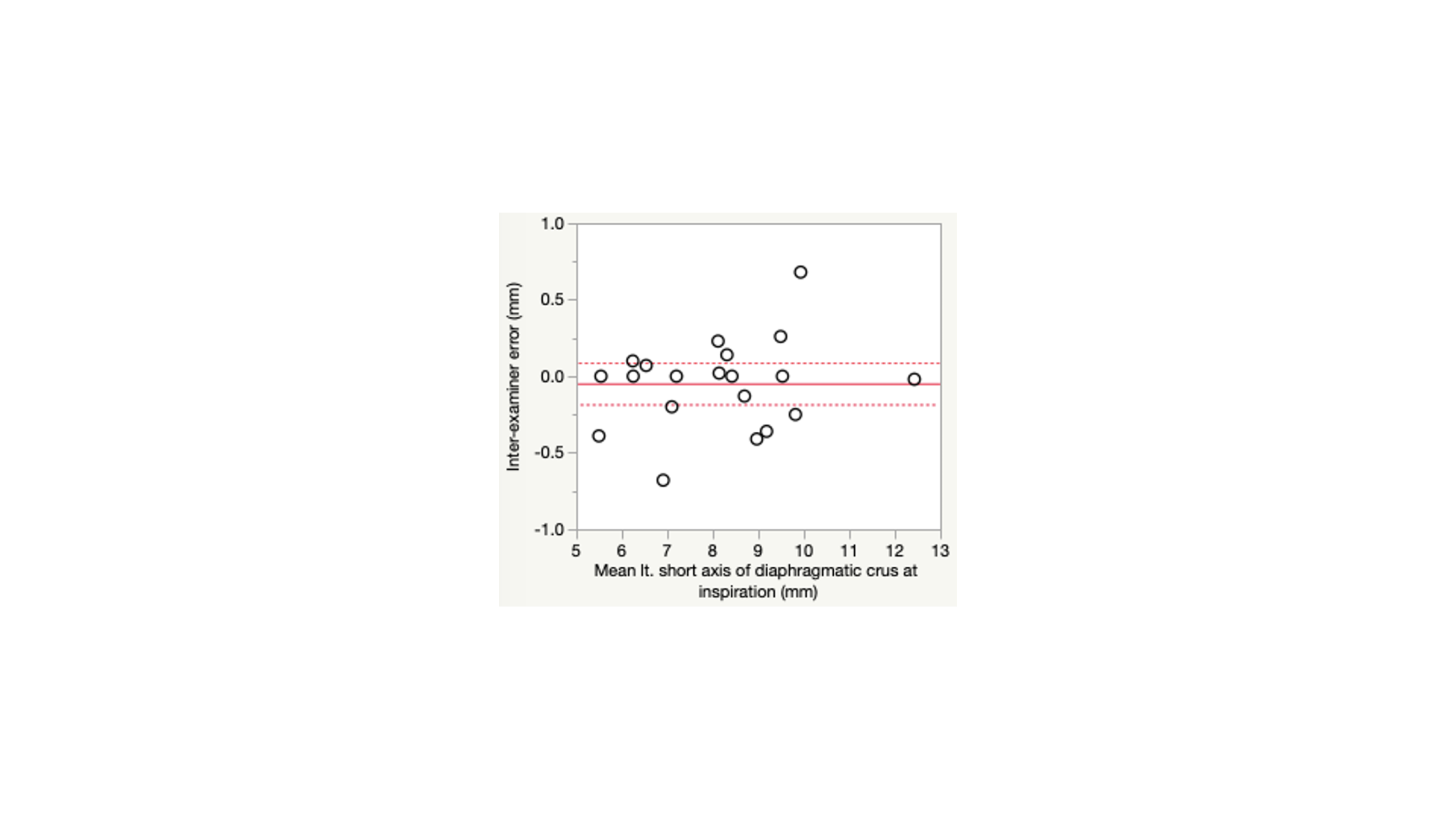
**

**Supplemental Fig. 1–**Reproducibility of measuring left short axis of diaphragmatic crus at inspiration assessed with Bland-Altman analysis. The mean and the difference of measurements by the two examiners are plotted. The mean difference did not deviate significantly from zero, and inter-examiner error was small.


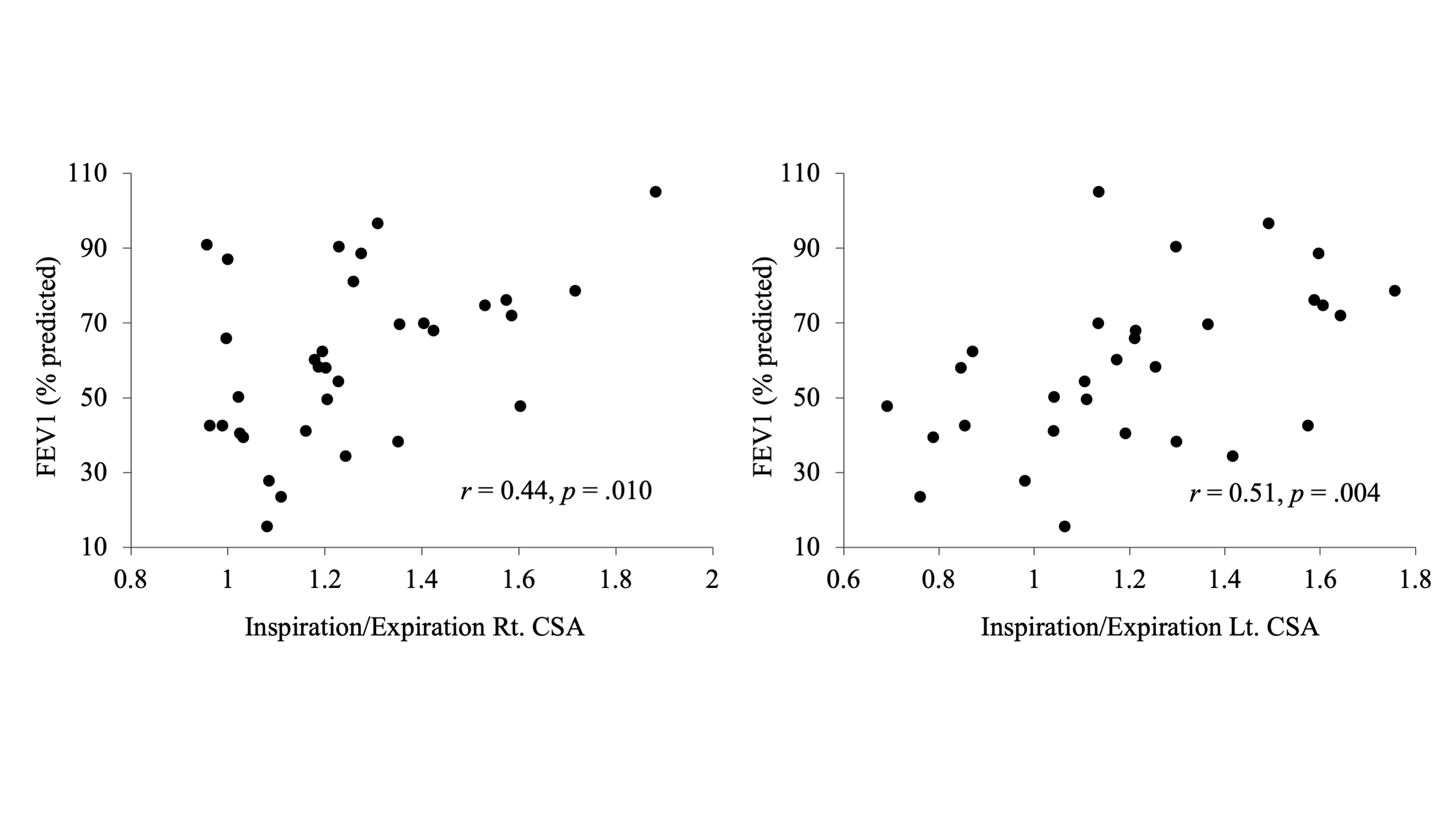


**Supplemental Fig. 2–**Positive correlation between the rate of change in the cross-sectional area of the bilateral diaphragmatic crura after expiration and pulmonary function tests.
